# Supplementary material for: Studying stochastic systems biology of the cell with single-cell genomics data
Source: bioRxiv. 2023 May 29:2023.05.17.541250. Preprint. [Version 2] doi: 10.1101/2023.05.17.541250 (PMC10245677; doi:10.1101/2023.05.17.541250)
Supplement: Supplement 3 [file NIHPP2023.05.17.541250v2-supplement-3.pdf]

bioRxiv preprint doi: https://doi.org/10.1101/2023.05.17.541250; this version posted May 29, 2023. The copyright holder for this preprint (which was not certified by peer review) is the author/funder, who has granted bioRxiv a license to display the preprint in perpetuity. It is made available under aCC-BY 4.0 International license.

Supplementary Tables

Term	Interpretation
$H_{is}(t)P(i,\mathbf{x},\mathbf{y},t)$	Transition from categorical state i to s
$c_{i0}\left[(x_i+1)P(s,x_i+1,\mathbf{y},t)-x_iP(s,\mathbf{x},\mathbf{y},t)\right]$	Degradation of discrete species i
$c_{ij}\left[(x_i+1)P(s,x_i+1,x_j-1,\mathbf{y},t)-x_iP(s,\mathbf{x},\mathbf{y},t)\right]$	Conversion of discrete species i to j
$Q^d_{ii}\left[(x_i-1)P(s,x_i-1,\mathbf{y},t)-x_iP(s,\mathbf{x},\mathbf{y},t)\right]$	Autocatalysis of discrete species i
$Q^d_{ji}\left[x_iP(s,x_j-1,\mathbf{y},t)-x_iP(s,\mathbf{x},\mathbf{y},t)\right]$	Catalysis of discrete species j by i
$\alpha^d_{s,\omega}(t)\left[\sum_{\mathbf{z}}p^d_{s,\omega}(\mathbf{z},t)P(s,\mathbf{x}-\mathbf{z},\mathbf{y},t)-P(s,\mathbf{x},\mathbf{y},t)\right]$	Bursty production of discrete species
$-C^{cc}_{ji}\frac{\partial}{\partial y_j}\left[y_iP(s,\mathbf{x},\mathbf{y},t)\right]$	Increase in continuous species j proportional to level of continuous species i
$\sigma_i^2\frac{\partial^2}{\partial y_i^2}\left[y_iP(s,\mathbf{x},\mathbf{y},t)\right]$	Square-root noise in continuous species i
$-\alpha^c_{s,i}(t)\frac{\partial P(s,\mathbf{x},\mathbf{y},t)}{\partial y_i}$	Drift in continuous species i
$\alpha^c_{s,\omega}(t)\left[\int_{\mathbf{z}}p^c_{\omega}(\mathbf{z})P(\mathbf{y}-\mathbf{z},t)d\mathbf{z}-P(\mathbf{y})\right]$	Bursting in continuous species
$C^{cd}_{ji}\left[y_iP(x_j-1,\mathbf{y},t)-y_iP(\mathbf{x},\mathbf{y},t)\right]$	Production of discrete species j proportional to level of continuous species i
$-C^{dc}_{ji}x_i\frac{\partial P(\mathbf{x},\mathbf{y},t)}{\partial y_j}$	Drift in continuous species j proportional to number of discrete species i

Table S1 Components of the full master equation.

Identifier	Source	Technology	Species	Tissue or culture
`pbmc_1k_v3`	[173]	10x v3 sc	*H. sapiens*	peripheral blood
`heart_1k_v3`	[174]	10x v3 sc	*M. musculus*	heart
`neuron_1k_v3`	[175]	10x v3 sc	*M. musculus*	neurons
`desai_dmso`	[116]	10x v2 sc	*M. musculus*	cultured embryonic stem cells treated with DMSO
`pbmc_1k_v2`	[176]	10x v2 sc	*H. sapiens*	peripheral blood technical replicate of `pbmc_1k_v3`
`brain_nuc_5k_v3`	[177]	10x v3 sn	*M. musculus*	neurons

Table S2 Datasets used for empty droplet analysis (sc: single-cell; sn: single-nucleus).

Table S3 Genes discovered to be overdispersed ($\sigma^2 > 2\mu$) in empty droplets for each dataset in Table S2. [Table provided in external spreadsheet]

Table S4 Genes discovered to be overdispersed ($\sigma^2 > 2\mu$) in empty droplets for the `neuron_1k_v3` and `desai_dmso` datasets, with function annotations. [Table provided in external spreadsheet]
